# Feasibility and Potential Effectiveness of a Smartphone Zero-Time Exercise Intervention for Promoting Physical Activity and Fitness in Patients With Coronary Heart Disease: A Pilot Randomized Controlled Trial

**DOI:** 10.3389/fpubh.2022.865712

**Published:** 2022-07-14

**Authors:** Noel P. T. Chan, Agnes Y. K. Lai, Hau K. Choy, Derek Y. T. Cheung, Alice N. T. Wan, Victor Y. H. Cheng, Ka Y. Chan, Yuk K. Lau, Chi Y. Yung, George O. C. Cheung, T. H. Lam

**Affiliations:** ^1^School of Nursing, The University of Hong Kong, Pokfulam, Hong Kong SAR, China; ^2^School of Nursing and Health Studies, Hong Kong Metropolitan University, Hong Kong, Hong Kong SAR, China; ^3^Faculty of Medicine, Poznon University of Medical Sciences, Poznan, Poland; ^4^Aberdeen Kai Fong Welfare Association Services Centre, Aberdeen, Hong Kong SAR, China; ^5^Division of Cardiology, Department of Medicine and Geriatrics, Pok Oi Hospital, New Territories, Hong Kong SAR, China; ^6^Intensive Care Unit, Hong Kong Sanatorium Hospital, Happy Valley, Hong Kong SAR, China; ^7^Private Practice, Hong Kong, Hong Kong SAR, China; ^8^Division of Cardiology, Department of Medicine, Ruttonjee and Tang Shiu Kin Hospitals, Wan Chai, Hong Kong SAR, China; ^9^School of Public Health, The University of Hong Kong, Pokfulam, Hong Kong SAR, China

**Keywords:** zero-time exercise, physical activity, coronary heart disease, e-message, smartphone apps, randomized controlled (clinical) trial, intervention, physical fitness

## Abstract

**Background:**

Zero-time Exercise (ZTEx), a simple strength- and stamina-enhancing physical activity (PA) requiring no extra equipment, can potentially increase PA and fitness. This pilot trial examined the feasibility and potential effectiveness of a smartphone ZTEx intervention to promote PA and fitness in patients with coronary heart disease (CHD).

**Methods:**

A parallel-group assessor-blinded pilot randomized controlled trial was conducted on Chinese patients with stable coronary heart disease (CHD) in three cardiology clinics. The experimental group received a 15-min brief individual face-to-face session and a 12-week ZTEx instant messaging with 28 picture e-messages and a smartphone ZTEx application (ZTExApp). The control group received the same duration of individual session and number and format of e-messages, but the content was healthy eating and breathing exercise. The feasibility was assessed based on: attrition rate, usage, response rate and perception of the intervention. The outcome evaluation included primary outcome (PA), fitness, exercise self-efficacy and intention, perceived happiness and health, and quality of life. A linear mixed model was used with intention-to-treat analysis adjusting for sex, age and baseline values. A semi-structured interview was conducted to collect feedback from the experiment group.

**Results:**

One hundred thirty-nine patients (mean age 59.8 ± 6.6; 71.2% male) were randomized to the experimental group (*n* = 70) or control group (*n* = 69), and 80% (56/70) and 82% (57/69) of patients completed the 12-week follow-up assessment, respectively. The attrition rate was 18.7%. The experimental group reported that ZTEx was feasible to integrate PA into their daily life and appreciated the picture e-messages, and 95% of them sent feedback to us, but only 19.6% (13/70) of the participants entered their PA information into the e-diary of the ZTExApp. The experimental group had a significantly greater increase in time spent walking [mean difference (95% CI): 155.3 (10.1, 300.4), *P* = 0.04, Cohen's *d* = 0.34] than the control group.

**Conclusions:**

This pilot study showed using a brief ZTEx face-to-face session with picture e-messages empowered patients with CHD to integrate PA into daily life. Future definitive trials with a longer follow-up and a more user-friendly ZTExApp interface are necessary to determine the effectiveness of the smartphone ZTEx intervention in enhancing PA and related outcomes.

**Trial Registration:**

The research protocol was registered at the Hong Kong University Clinical Trials Registry (HKUCTR) on 22 Jul 2016 (Study identifier: HKUCTR-2165) and was also retrospectively registered at the National Institutes of Health (identifier number: NCT03464331) on 14 March 2018.

## Introduction

Coronary heart disease (CHD) is the third leading cause of mortality worldwide and is associated with 17.8 million deaths annually (1). In Hong Kong, CHD is the most common type of heart disease, accounting for 7.7% of all registered deaths in 2019 (2). The American Heart Association highlighted the deleterious association between physical inactivity and the morbidity and mortality of heart disease (3). Regular physical activity (PA) increases myocardial oxygen demand, which acts as a stimulus to increase coronary blood flow and myocardial oxygen supply, thus reducing myocardial infarction and angina, improving endothelial and coronary smooth muscle function and coronary vasodilation, and decreasing CHD morbidity and mortality (4). Reviews also showed that physical activity interventions improved exercise capacity, enhanced physical functioning, and reduced cardiovascular risks (5). A higher impact on PA behaviors was reported in theory-based PA interventions than no theory-based physical activity interventions (6). However, uptake of and adherence to a physically active lifestyle remain problematic. Despite the well-known benefits of regular physical activity, patients with CHD engage in little physical activity (7), the most common barrier to regular physical activity are lack of time and the belief that regular exercise is time-consuming (8).

Our team used Zero-time exercise (ZTEx), a new approach to integrating simple strength- and stamina-enhancing physical activity into daily life, which does not require extra time, money, and equipment and can be done anytime, anywhere, and by anybody. ZTEx uses a foot-in-the-door approach to build exercise self-efficacy and habits, starting with small steps and simple and easy-to-do exercises (9). Pedaling while sitting and standing on one leg while waiting for bus are the examples of ZTEx and more examples are shown in our YouTube videos (https://www.youtube.com/user/familyhk3h/videos). This approach is consistent with American Physical Activity Guidelines that moving more and sitting less is beneficial for nearly everyone and some physical activity is better than none (10). We have applied ZTEx interventions to various clinical and community-based programs for different populations (e.g., low-income families, parents, children, elderly, and patients with insomnia), with consistently positive results in increasing exercise motivation, PA, and fitness (11–16).

With the advancement of digital health technologies, phone apps and messaging as mHealth inventions are increasingly popular and have shown more evidence of efficacy in the health care sector. We found 12 randomized controlled trials (RCTs) of the internet- and mobile-based phone delivered interventions to improve PA in CHD patients (17–28). Five RCTs used PA-related text messages only (18, 20, 21, 23, 24), and three of them reported enhanced PA (18, 23, 24). The other five RCTs using text messages with other interventions (e.g., web-based video messages, telephone coaching, emails, tracking technologies, online discussion) (22, 25–28), and the remaining 2 RCTs using PA-related Apps also showed increased PA (17, 18).

Hong Kong has extensive smartphone penetration (85.8% in 2017) (29), and strong evidence supports the value of mobile health intervention in CHD. Mobile text reminders were crucial components in behavioral intervention with the aim of an individual receiving repetitive information over time. A review of 10 RCTs reported mobile text reminder interventions showing trends toward a significant improvement in medication adherence behavior, but no RCT on PA adherence (30). We also posited that habitual physical inactivity is the major inertia of having regular physical activity. This could be overcome by simple repeated reminders that might affect the action of neural memory units for behavior and increase the desired adherence. Such anti-inertia reminders (AIR) could be easily sent to participants through mobile messaging at no or low costs. Although picture e-messages are simple visual communication that could benefit all audiences, especially those with lower literacy, we found no RCT using picture e-messages only, with or without an app, to promote PA in patients with CHD.

This was the first pilot trial to examine the feasibility and potential effectiveness of a smartphone ZTEx intervention promoting PA and fitness in CHD patients with process and outcome evaluation. We conducted mixed-method evaluation, including pre-and post-intervention questionnaire surveys and semi-structured individual interviews to examine the intervention and its components quantitatively and qualitatively. We integrated quantitative and qualitative findings in a triangulation fashion to enrich our understanding of the response. We anticipated that the experimental group would spend more time on PA and have higher exercise self-efficacy and intention in relation to ZTEx, perceived happiness and health, a better quality of life, and physical fitness than the control group at the 12-week follow-up.

## Methods

### Study Design and Settings

A 12-week pilot, two-arm, parallel-group (1:1) RCT was conducted in three cardiology out-patient clinics of three regional hospitals under the Hong Kong Hospital Authority from October 2016 to August 2018. This study was approved by The Institutional Review Board of The University of Hong Kong/Hospital Authority Hong Kong West Cluster (reference number: UW16-363). All participants provided written informed consent before joining the study. The study was registered at the Hong Kong University Clinical Trials Registry (HKUCTR) on 22 Jul 2016 (Study identifier: HKUCTR-2165) and US National Institutes of Health (ClinicalTrials.gov Identifier: NCT03464331) on 14 March 2018.

### Participants

The inclusion criteria were: (i) aged 18–69 years with stable CHD, i.e., asymptomatic or symptomatic but controlled by medications; (ii) no revascularisation performed in the past 3 months; (iii) able to walk for at least 15 min independently with normal speed without breathlessness; (iv) a smartphone user; and (v) never used the smartphone ZTEx application (ZTExApp). The ZTExApp could be used on Andriod and Apple smartphones. The exclusion criteria were: (i) having any medical conditions that could limit their ability to perform moderate-intensity PA; (ii) performing moderate/vigorous PA more than 150 min/week at the time of recruitment; and (iii) participating in a cardiac rehabilitation programme. Since this was a pilot study for a group of patients with coronary heart disease using the context of ZTEx to ask patients to do PA according to their ability and schedules and we would like to ensure patient safety, thus we prefer to recruit patients with more stable conditions in general. We directly asked the potential participant's perceived physical activity level: “In the past seven days, did you perform moderate/vigorous performing moderate/vigorous PA for more than 150 min/week?” We also defined moderate and vigorous PA to the participants; when they were doing the moderate-intensity activity, they could talk but not sing. When they were doing vigorous-intensity exercise, they could not say more than a few words without pausing for a breath during the activity.

### Intervention

#### The Experimental Group

The experimental group received a brief ZTEx intervention designed by our multidisciplinary team, including a doctor, nurses and an exercise trainer. The intervention was grounded on the Health Action Process Approach model focusing on the psycho-cognitive factors across behavior change in two distinctive stages, including the motivational and the volitional stages. In the motivational phase, we enhanced the beliefs related to the risks of physical inactivity (risk perceptions), the realistic outcome expectation of having regular PA (outcome expectancies) and the confidence to perform simple strength- and stamina-enhancing lifestyle-integrated PA (self-efficacy). The volitional phase mainly includes planning and maintaining health behavior changes (31). This framework has been applied in some studies for CHD patients (32, 33). Besides, based on the concept of a care bundle to maximize the intervention effect, the combination of several simple interventions could achieve some measurable effects (34). We delivered a bundle of interventions, including a brief 15-min individual face-to-face ZTEx-related information session (Part 1), a 12-week ZTEx picture e-messages *via* WhatsApp as exercise reminders (Part 2), and a smartphone ZTExApp (Part 3).

In part 1, we first briefly discussed with the participants the risk factors related to CHD and ways of reducing the risks. We introduced the concepts of ZTEx and its health benefits, demonstrated different poses of ZTEx and invited the participants to perform the ZTEx together. We used a foot-in-the-door approach and emphasized the importance of safety to encourage starting from a simple and easy-to-do exercise first with a gradual increase. The participants were encouraged to do any type [simple strength-and stamina-enhancing, light (e.g., brisk walking) and moderate PA (e.g., Climbing stairs)] for any duration, based on their health condition and personal limitations. Supplementary Table 1 shows the steps and aims of the brief individual face-to-face ZTEx-related session.

In part 2, we designed a series of 28 picture e-messages to enhance disease and physical activity knowledge (e.g., benefit of having regular PA) and self-efficacy (e.g., recommend starting from simple exercise first) and suggest home-based exercises (e.g., foot paddling while sitting) and as anti-inertia reminders to remind patients to be active. Patients are encouraged to send a simple message (e.g., emoji) after receiving instant messages to encourage 2-way communication. Supplementary Table 2 shows the theory-based picture PA e-messages with an English translation and the goals of each message. These were sent *via* WhatsApp with a tapered schedule of daily picture e-messages in week one, three times a week from week 2 to 6, and then once a week from week 7 to 12. The declining frequency of engagement would be more acceptable for participants and feasible for further implementation in real clinical practice (by saving costs). It would allow participants to become less dependent toward the end and then independent after the intervention.

In part 3, we introduced the ZTExApp during the individual session, assisted the participants in downloading the ZTExApp to their smartphones, and taught them to use the ZTExApp. Supplementary Figure 1 shows the components of the ZTExApp. The ZTExApp included (i) ZTEx information and the six main components of body fitness (body composition, balance, flexibility, strength, endurance and cardiopulmonary performance), (ii) examples of enhancing body fitness (e.g., handgrip training to strengthen the upper limb muscle, foot peddling to strengthen the low limb muscle strength, and walking to enhance cardiopulmonary performance), (iii) fitness self-assessment tests (e.g., lower limb strength measured by 30-s Chair Stand Test), and (iv) a built-in e-diary for goal setting and self-monitoring.

#### The Control Group

The duration of the individual face-to-face session and the number of picture e-messages for the control group were the same as the experimental group. In part 1, the control group received information on risk factors of cardiovascular disease, ways and benefits of healthy eating (e.g., low-fat, -salt, and -sugar intake), and benefits and skills of breathing exercises (such as muscle relaxation, stress and anxiety reduction, better sleep quality, etc.). The interventionist practiced breathing exercises with the participants together to increase their self-efficacy. Supplementary Table 1 shows the steps and aims of the brief healthy eating and breathing exercise information session. In part 2, the control group received 28 picture e-massages with the same tapered schedules, but the contents were about healthy eating and breathing exercises. Supplementary Table 2 shows the theory-based picture PA e-messages with an English translation and the goals of each message. The participants were asked to record their PA in a paper diary.

Because of resource constraints, we only gave Fitbit HR (Fitbit) to the first 50 recruited participants (*n* = 25/group) to measure daily steps walked during the 12-week study period. We helped download and synchronize the Fitbit App to the participants' smartphones and gave a brief introduction on how to use the Fitbit device. The participants in both groups were instructed to respond to our picture e-messages by returning an emoji and asking questions *via* WhatsApp.

#### The Feasibility

The feasibility of the intervention was assessed based on the following indicators: attrition rate and the usage, response rate and perception of the intervention components. The attrition rate was the proportion of participants who did not complete the 12-week assessment. The usage of ZTExApp was measured by the proportion of participants using the e-diary platform in the ZTExApp to record their physical activity performance. Such usage was considered as one of the indicators of adherence to ZTEx intervention in the experimental group. The response rate of picture e-messages was measured by the proportion of participants who had provided feedback with “emoji” through WhatsApp to us after receiving our picture e-messages. The perception of intervention components was assessed by the qualitative feedback from the participants at the completion of the trial.

#### Outcome Evaluation

##### Physical Activity

The time spent walking and on moderate/vigorous PA (min/week) in the past 7 days was measured by the Chinese version of the International Physical Activity Questionnaire (IPAQ-C), which had high internal reliability with an intra-class correlation coefficient of 0.79 (35). The proportion of participants who performed moderate physical activity was calculated by the number of participants who performed moderate physical activity at least 1 day during the study period divided by the recruited. The proportion of participants who performed vigorous physical activity was calculated by the number of participants who performed moderate physical activity at least 1 day during the study period divided by the recruited. All the Fitbit subgroup participants were asked to wear Fitbit except while bathing, swimming and sleeping. The number of daily steps walked as measured by Fitbit throughout the 12-week study.

##### Exercise Self-Efficacy and Intention

The four outcome-based statements were used to assess the experimental group only on their knowledge of self-efficacy, outcome expectancy and plan to perform ZTEx. The four statements were: (i) “I understand the concept of ZTEx”; (i) “If I can do zero-time exercise regularly, I believe I can as it improves my health and prevents illness”; (iii) “I am confident that I can always do zero-time exercise, even if I need to learn how to build this habit.” and (iv) “I intend to do zero-time exercise regularly.” The response to each item was on a scale from “0 = strongly disagree” to “10 = strongly disagree,” with higher scores indicating better outcomes (15).

##### Body Composition and Physical Function

Based on standardized protocols, body composition, body weight (36), per cent of body fat (37), waist circumference (38), and muscle strength were measured. Handgrip strength was measured by a dynamometer (39), and lower limb strength was assessed using a 30-s Chair Stand Test to record the total number of stands within 30 s (40).

##### Perceived Happiness and Health

Perceived happiness and health were assessed by asking two direct questions: “Do you think you are happy?” and “Do you think you are healthy?” The response was from “0 = very unhappy/unhealthy” to “10 = very happy/healthy,” with higher scores indicating better outcomes (9).

##### Quality of Life

A World Health Organization Quality of Life Instrument-Short Form (WHOQOL-BREF) assessed the quality of life with a 5-point Likert scale. The WHOQOL-BREF included domains of physical health (seven items), psychological (six items), social relationships (three items) and environment (eight items). The internal consistency with Cronbach's alpha coefficients ranged from 0.70 to 0.77 (41).

##### Qualitative Feedback on the ZTEx Intervention

Fifteen-min semi-structured face-to-face individual interviews collected participants' feedback on the ZTEx intervention. Supplementary Table 3 shows the questions of the semi-structured interview guide. Ten participants from the experimental group were randomly selected when they returned their Fitbit to us and after answering the questionnaire and performing a fitness assessment at the 12-week follow-up. All interviews were conducted in Cantonese in the three cardiology clinics by trained research staff, audio-recorded and transcribed verbatim. The semi-structured questions were designed to collect data on participants' satisfaction with, barriers to, and attitudes toward using the ZTExApp, and their feedback on the ZTEx picture e-messages and Fitbit use.

### Sample Size

The sample size calculation was based on feasibility and generated estimates to guide future main trials' statistical power and sample size calculation. By repeated measures, ANOVA (*F*-test) in GPower 3.1, assuming two effect sizes of PA min per week as 0.25 and 0.5, 5% significance, measurements at baseline and 12-week follow-up, at least 50 patients in each arm were needed to achieve a statistical power of 80% with a 20% attrition rate at the 12-week follow-up.

### Randomization

A randomization sequence was generated using Microsoft Excel with a 1:1 allocation and random block sizes of 2 and 4 by an independent research staff member. Because of resource constraints, only the first 50 participants (*n* = 25/group) were given Fitbit to measure the daily steps walked during the 12-week study. The participants in the Fitbit subgroups were randomly selected for the face-to-face interview (*n* = 10/group). Sequentially numbered, opaque, sealed envelopes (SNOSE) were used to ensure allocation concealment. After obtaining informed consent and completing the baseline assessment, the research staff opened one envelope according to the serial number sequence and assigned the participant to either the experimental or control group with or without a Fitbit. Blinding of patients and research staff administering face-to-face sessions was not practicable because the intervention encouraged behavioral changes, but outcomes assessors were blinded.

### Statistical Analysis

Analyses were conducted using SPSS version 26. All tests were two-sided, with *P* < 0.05 indicating statistical significance. Participants' demographic characteristics, including sex, age, body composition, marital status, working status and smoking status, were examined by the Chi-square test. Sex and age were considered potential confounders. All between-group analyses were adjusted by sex, age and baseline values. Intention-to-treat (ITT) analysis was conducted for all participants who joined the trial with the missing values replaced by baseline values of the outcome variables. Per-protocol (PP) analysis of those participants who completed the 12-week assessment was conducted as a sensitivity analysis to determine the robustness of the ITT analysis results.

Within-group comparisons were examined by paired *t*-test for continuous variables and the likelihood ratio of McNemar's test for categorical variables. Between-group differences in the baseline characteristics of two groups were compared by independent *t*-test and chi-square test for continuous and categorical variables. A linear mixed model was used to compare the differences in time spent walking on moderate and vigorous PA, the number of steps walked, body fitness performance, perceived happiness, health, and quality of life. Logistic regression was used to compare differences in the proportion of participants who performed moderate and vigorous PA in the past 7 days between two groups at a 12-week follow-up.

Participants with at least 75% (9 weeks) Fitbit wear-time requirement were included in the between-group steps walked comparison analysis. The last-observation-carried-forward (LOCF) imputation method was used for missing data. The intervention effect in Fitbit subgroups on walking time was examined by including an interaction term (Fitbit × group) in the linear mixed model analysis. An effect size (Cohen's *d*) of 0.2, 0.5 and 0.8 or above was considered a small, medium and large effect.

All qualitative interviews were audiotaped and transcribed verbatim in Chinese. Transcripts were analyzed by thematic content analysis (42). The mixed-method triangulation approach was used to corroborate the findings and complement the results in interpretation (43).

## Results

### Participants

**Figure 1:**
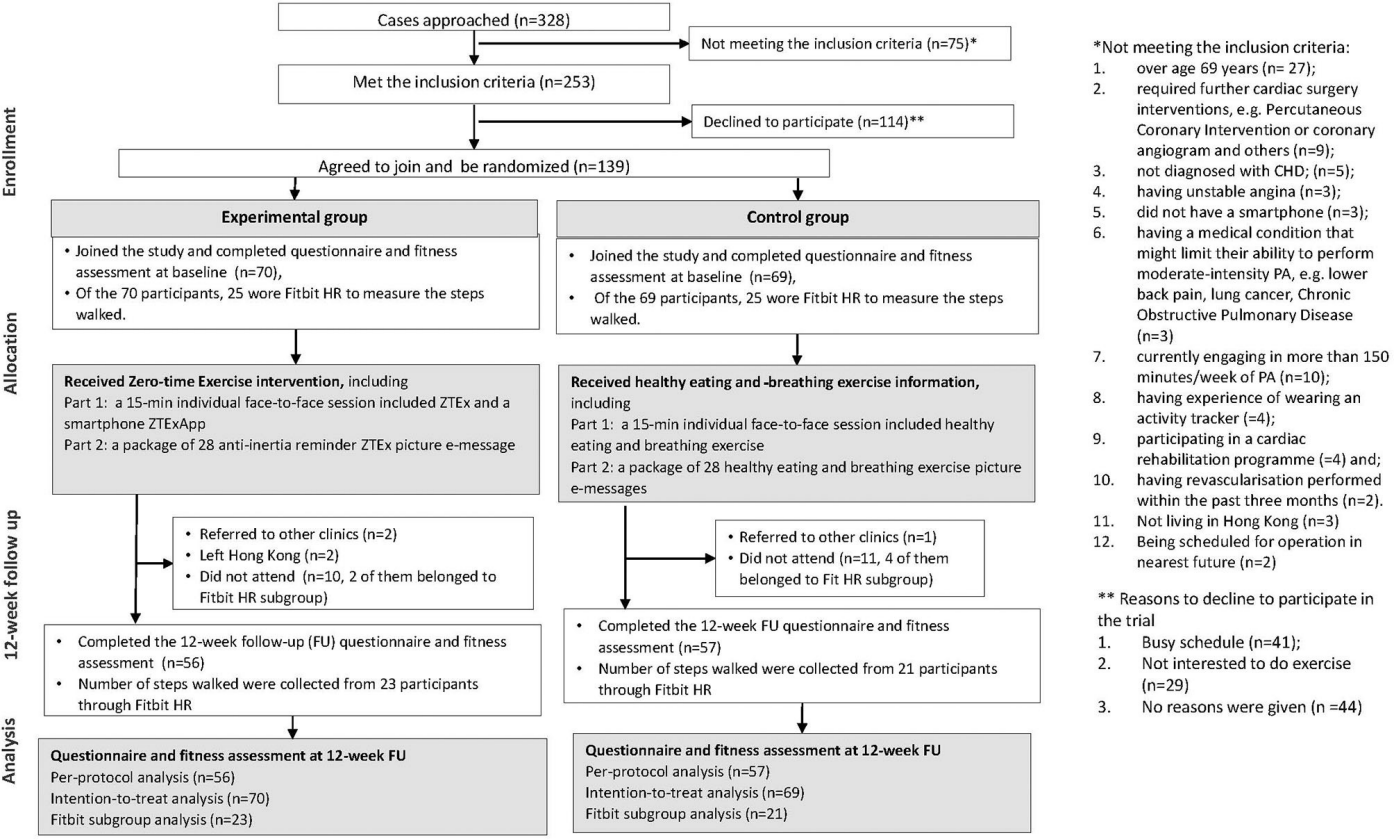
The flow of participants (The CONSORT diagram).

A total of 328 patients were approached, 75 and 114 of them did not meet the inclusion criteria and declined to join the trial, respectively. One hundred thirty-nine (mean age 59.8 ± 6.6 years; 71.2% male) participants agreed to join and were randomized to the experimental (*n* = 70) or control group (*n* = 69). In the experimental group, two patients were referred to other clinics, and two left Hong Kong. Ten patients did not perform the 12-week assessment; two of them belonged to the Fitbit subgroup. In the control group, a patient was referred to other clinics. Eleven patients did not attend the follow-up; four of them belonged to the Fitbit subgroup. A total of 26 patients were lost to follow-up and 113 patients [experimental (*n* = 56/70, 80%), control groups (*n* = 57/69, 83%)] completed the 12-week follow-up assessment. The attrition rate was 18.7%. Figure 1 shows the flows of participants (CONSORT diagram). No harm and adverse effects were reported. Table 1 shows that the mean age was 60 years, and no substantial differences in baseline characteristics, except for sex with fewer men in the experimental than in the control group (62.9 vs. 79.7%, *P* = 0.03).

**Table 1:** Characteristics of the participants at baseline (*n* = 139).

	**Experimental group** **(*n* = 70)**	**Control group** **(*n* = 69)**	***P*** **-Value**
	***n*** **(%)**	***n*** **(%)**	
**Sex**			
Female	26 (37.1)	14 (20.3)	0.03^*^
Male	44(62.9)	55 (79.7)	
**Age, years** ^**#**^	59.8 ± 6.4	59.8 ± 6.8	0.94
**Body Mass Index (kg/m** ^**2**^ **)** ^**#**^	26.1 ± 3.8	26.7 ± 3.4	0.30
**Marital status**			
Single/divorced	12 (17.1)	15 (21.7)	0.49
Married	58 (82.9)	54 (78.3)	
**Working status**			
Retired, unemployed, or working part-time	38 (54.3)	35 (50.7)	0.67
Working full-time	32 (45.7)	34 (49.3)	
**Smoking status**			
Never smokers	49 (70.0)	41 (59.4)	0.33
Smokers and ex-smokers	20 (29.0)	24 (34.8)	
Missing data	1 (1.4)	4 (5.8)	

# *Presented as mean ± SD*.

Between-group comparison:

* *P < 0.05*.

### Outcomes

#### Physical Activity

**Figure 2:**
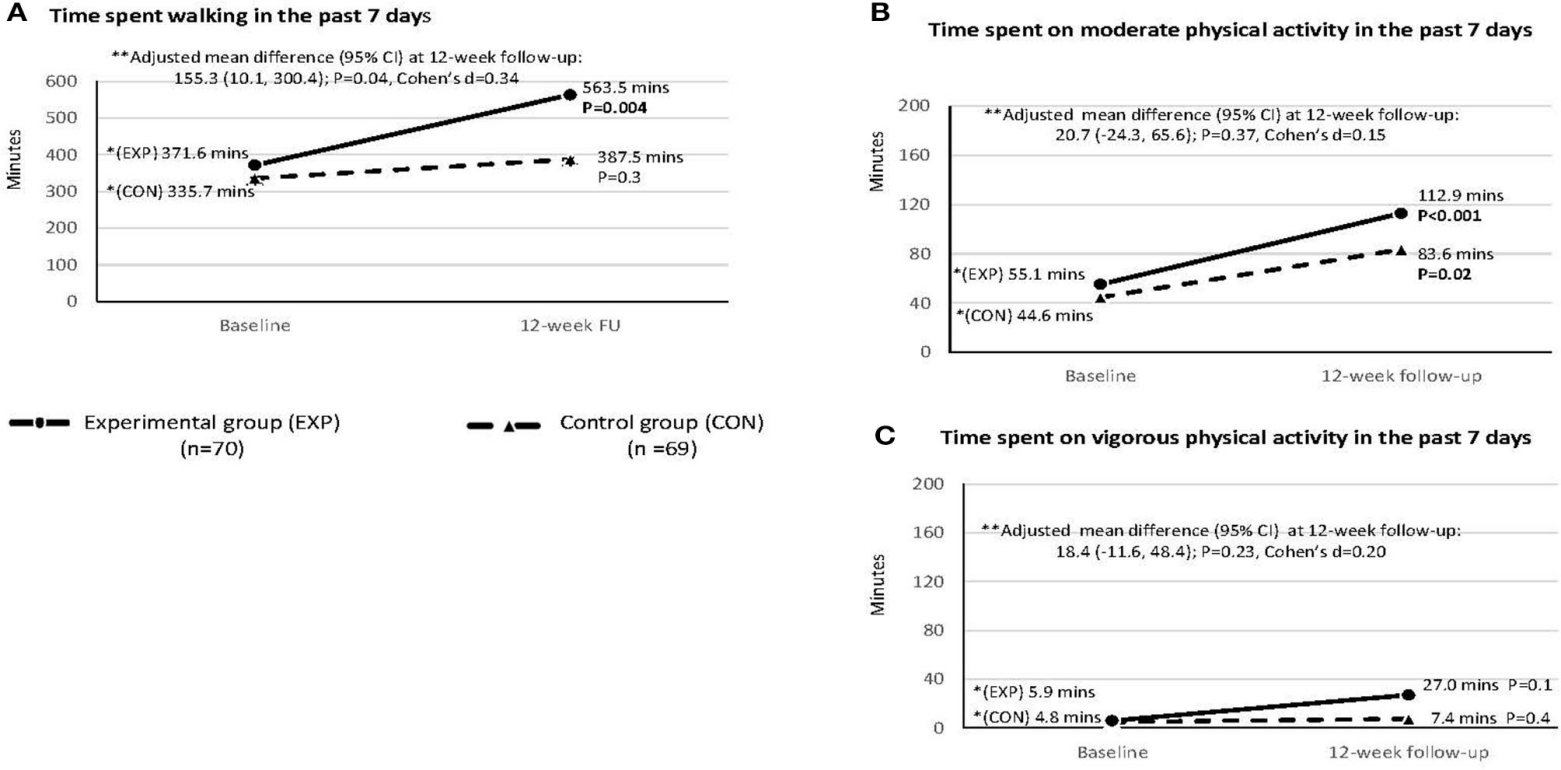
Time spent walking and moderate and vigorous physical activities in the 7 days at baseline and 12-week follow-up: Intention to treat analysis (*n* = 139). This was measured by the short form of International Physical Activity Questionnaire—Chinese version. *Within-group comparison was done by paired sample t-test. **Between-group comparison by linear mixed model with adjustment of age, sex and baseline values; Cohen'd effect size ~0.2, medium effect ~0.5, and large effect ~0.8 or above.

Figure 2 shows that the experimental group spent significantly more walking time measured by (IPAQ-C) than the control group (563.5 vs. 387.5 min respectively) at the 12-week follow-up [adjusted mean difference (95% CI): 155.3 min (10.1, 300.4), *P* = 0.04, Cohen's *d*: 0.34; Panel A]. Time spent on moderate [adjusted mean difference (95% CI): 20.7 (−24.3, 65.6), *P* = 0.37, Cohen's *d* = 0.15; Panel B] and vigorous PA [adjusted mean difference (95% CI): 18.4 (−11.6, 48.4), *P* = 0.23, Cohen's *d* = 0.20; Figure 2C] at the 12-week follow-up showed no significant difference between two groups.

**Figure 3:**
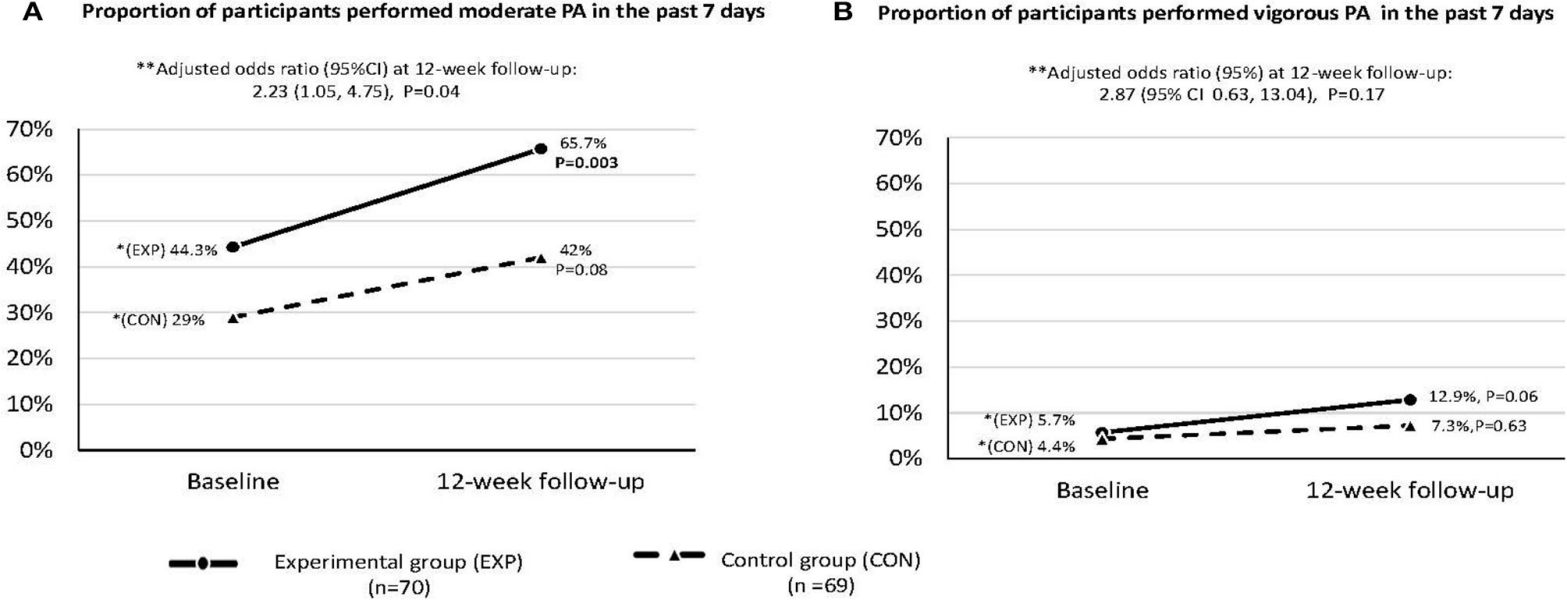
The proportion of participants who performed moderate and vigorous physical activities in the past 7 days at baseline and 12-week follow-up: Intention-to-treat analysis (*n* = 139). This was measured by the short form of International Physical Activity Questionnaire—Chinese version. *Within-group comparison was done by McNemar test. **Between-group comparison by logistic regression with adjustment of age, sex and baseline values.

Figure 3 shows that the proportion of participants in the experimental group engaging in moderate PA was significantly higher than the control group [65.7 vs 42.0%, adjusted odds ratio (AOR, 95% CI): 2.23 (1.05, 4.75), *P* = 0.04]. For vigorous PA, the adjusted AOR (95% CI) was 2.87 (0.63, 13.04) but was not significant (*P* = 0.17).

Six participants (experimental group, *n* = 2; control group, *n* = 4) who only used Fitbit for less than a week were excluded from the Fitbit subgroup. The mean daily steps walked in the experimental group (*n* = 23; 10,206 steps) was 26.5% greater than that of the control group (*n* = 21; 8,068 steps) but the difference was marginally insignificant [adjusted mean difference (95% CI): 2,138 (−137, 4,411), *P* = 0.07]. No significant interaction (Fitbit x group) was observed (*P* = 0.26).

#### Exercise Self-Efficacy and Intention

Table 2 shows no significant increase in knowledge (a marginally insignificant increase from 6.8 to 7.4, *P* = 0.07, Cohen's *d* = 0.23) and self-efficacy, outcome expectancy, and plan to do ZTEx in the experimental group. The control group was not asked these four questions.

**Table 2:** Exercise self-efficacy and intention, physical fitness, perceived happiness and health, and quality of life of the experimental and control groups at baseline and 12-week follow-up by intention-to-treat analysis (*n* = 139).

	**Experimental group (*****n*** **= 70)**				**Control group (*****n*** **= 69)**				**Between-group comparison at 12-week follow-up**		
	**Baseline**	**12-week follow-up**	**Within-group difference**		**Baseline**	**12-week follow-up**	**Within-group difference**				
	**Mean ± SD**	**Mean ± SD**	***P*** **-Value**	**Cohen's *d***	**Mean ± SD**	**Mean ± SD**	***P*** **-Value**	**Cohen's *d***	**Adjusted mean difference (95% CI)**	***P*** **-Value**	**Cohen's *d***
**Exercise self-efficacy and intention**											
Knowledge of ZTEX	6.8 ± 2.8	7.4 ± 2.2	0.07	0.23	—	—	—	—	—	—	—
Self-efficacy of doing ZTEx	6.7 ± 2.3	6.7 ± 1.9	0.91	0.01							
Expectancy toward ZTEx	7.1 ± 2.3	7.5 ± 2.0	0.24	0.15	—	—	—	—	—	—	—
Plan on doing ZTEx	7.1 ± 2.3	6.9 ± 2.1	0.51	−0.08	—	—	—	—	—	—	—
**Physical fitness**											
**Body composition**											
Bodyweight, kg	70.2 ± 12.9	70.2 ± 12.8	0.98	0	73.5 ± 11.5	73.4 ± 11.4	0.53	−0.01	0.06 (−0.47, 0.60)	0.81	0.04
Body fat, %	30.6 ± 6.1	30.3 ± 6.6	0.30	−0.13	29.1 ± 5.5	29.0 ± 5.3	0.69	−0.05	−0.21 (−1.10, 0.68)	0.64	−0.07
Waist circumference, cm	92.5 ± 11.2	92.0 ± 10.9	0.13	−0.04	95.6 ± 9.6	95.7 ± 8.3	0.87	0.01	−0.89 (−2.20, 0.42)	0.18	−0.22
**Muscle strength**											
Handgrip test (right), kg	31.1 ± 23.2	27.5 ± 8.5	0.16	−0.21	29.0 ± 9.3	29.9 ± 9.9	0.17	0.09	−1.17 (−3.50, 1.17)	0.32	−0.16
Handgrip test (left), kg	26.1 ± 9.2	26.1 ± 8.3	0.98	0	27.5 ± 9.5	27.8 ± 9.7	0.60	0.03	−0.18 (−1.75, 1.40)	0.82	−0.04
30-s chair stand test, no. of stands	15.3 ± 6.8	16.9 ± 6.4	0.02^*^	0.25	15.3 ± 4.2	15.9 ± 5.0	0.20	0.14	0.86 (−0.66, 2.39)	0.27	0.18
**Perceived happiness and health**											
Perceived happiness	6.8 ± 1.6	7.2 ± 1.7	<0.001^***^	0.27	7.2 ± 2.0	7.4 ± 1.8	0.32	0.09	0.15 (−0.25, 0.54)	0.47	0.12
Perceived health	6.1 ± 1.9	6.1 ± 1.6	0.80	−0.03	6.1 ± 1.8	6.4 ± 2.1	0.28	0.15	−0.36 (−0.92, 0.20)	0.21	−0.21
**Quality of life**											
Physical health	14.4 ± 1.5	15.4 ± 1.9	<0.001^***^	0.57	14.3 ± 2.0	15.2 ± 2.0	<0.001^***^	0.43	0.23 (−0.34, 0.81)	0.42	0.13
Psychological	13.1 ± 1.5	15.3 ± 2.3	<0.001^***^	1.13	12.7 ± 2.0	14.9 ± 2.4	<0.001^***^	0.99	0.13 (−0.60, 0.85)	0.73	0.06
Social Relationships	15.0 ± 2.2	15.0 ± 2.2	0.79	0.03	15.1 ± 0.3	14.5 ± 2.3	0.017^*^	−0.26	0.68 (0.04, 1.31)	0.04^#^	0.35
Environment	15.2 ± 1.9	15.3 ± 1.9	0.50	0.08	15.1 ± 2.3	15.0 ± 2.1	0.65	−0.05	0.35 (−0.19, 0.89)	0.20	0.21

*ZTEx, Zero-time exercise refers to simple strength- and stamina-enhancing physical activity*.

*Exercise self-efficacy and intention regarding ZTEx using a scale of 0–10 for each question; higher scores indicate better outcomes*.

*Perceived happiness and health using a scale of 0–10; higher scores indicate better outcomes.*

*Quality of life using a WHO Quality of Life-Short Form (WHOQOL-BREF) with a 5-point Likert scale, higher scores indicate better quality of life.*

*Within-group comparison during the 12-week follow-up was increased by paired sample t-test;*

* *P < 0.05,*

*** *P < 0.001*.

*The between-group comparison was done by linear mixed model, adjusted for age, sex and baseline values;*

# *P < 0.05*.

*Effect size (Cohen's d): small, 0.2; moderate, 0.5; large, 0.8*.

#### Body Composition and Physical Function

Table 2 shows that the experimental group reported a significant increase in the number of *stands* in the 30-s Chair Stand Test (from 15.3 to 16.9, *P* = 0.02, Cohen's *d* = 0.25), while the control group showed no significant increase. The small between-group increase in the number of stands was not significant [adjusted mean difference (95% CI): 0.86 (−0.66, 2.39, *P* = 0.27, Cohen's *d* = 0.18)]. No significant within and between-group differences were found in other body composition (body weight, per cent of body fat and waist circumference) and other physical fitness outcomes.

#### Perceived Happiness and Health

Table 2 shows that the experimental group reported a significant increase in perceived happiness (from 6.8 to 7.2, Cohen's *d*: 0.27, *P* < 0.001), but not in the control group. The small between-group increase was not significant.

#### Quality of Life

Table 2 shows that the physical health and psychological domains scores significantly increased in both groups (all *P* < 0.001), but the between-group differences in changes were not significant. However, the social relationships score significantly decreased in the control group (*P* = 0.017, Cohen's *d*: −0.26), which was significantly lower by 0.68 (95% CI: 0.04, 1.31) than the experimental group (*P* = 0.04, Cohen's *d*: 0.35).

Per-protocol analyses were performed for those who completed the 12-week follow-up as a sensitivity analysis to ensure the robustness of the findings. Supplementary Table 4 shows the characteristics of participants who completed the 12-week follow-up with no significant difference between the two groups. Supplementary Table 5, Supplementary Figures 2, 3 show that the results of per-protocol analyses were similar to ITT analysis, except the increased proportion of participants who performed moderate PA was marginally insignificantly higher in the experimental group than in the control group [66.1 vs. 43.9%, adjusted odds ratio (AOR, 95% CI): 2.19 (0.98, 4.90), *P* = 0.06].

### The Feasibility

#### The Usage of the ZTEx App

Throughout the study period, 19.6% (13/70) of the participants in the experimental group entered their PA information into the e-diary in the ZTExApp on day 1, gradually dropped to 4% on day 4, and nobody entered their PA records in the e-diary thereafter. Due to the low usage of e-dairy, the collected information was insufficient for a valid analysis of the ZTEx intervention adherence. 17.4% (12/69) of the control group recorded their PA information on the paper diary on day 1, gradually dropped to 3% on day 18, and nobody entered their PA records in the paper diary thereafter.

#### Response to Picture e-Messages

Twenty-eight AIR picture e-messages were sent *via* WhatsApp to remind all participants in both groups to perform the briefed (taught) behaviors throughout the 12-week study. In the first week, a mean of 95% ranging from 94% (66/70) to 96% (67/70) of the experimental group sent “emoji” to us after receiving our picture e-messages. Although 2 participants withdrew from the study at week 2 and week 3, a high response rate of 94% (62/66) was maintained at week 12. Similarly, a high response rate was obtained in the control group too.

#### Perception of the ZTEx Intervention

The participants reported good acceptability of ZTEx intervention and commented that ZTEx was simple and convenient to do, especially walking. They liked ZTEx because it could be done anytime and anywhere. They felt good and reported perceived benefits after performing ZTEx. The quotes are as follows:

“I like to walk briskly. I feel good after started doing ZTEx.” (Female, 65 years old)

“I always perform ZTEx while using the computer, cooking or watching TV at home. It is simple and easy to do.” (Male, 57 years old).

“I don't feel breathless when climbing staircases after doing ZTEx regularly.” (Male, 59 years old)

“I try to increase my walking steps by increasing my walk time. When I was waiting for a bus, I did some ZTEx.” (Male, 57 years old)

“Walking is the most easy-to-do exercise, I walked to the bus stop every day.” (Male, 59 years old)

However, the participants reported barriers against ZTExApp use, including no internet access when going out, limited mobile data, and difficulty in reading the small smartphone screen even with the presbyopia glasses. Most participants in the experimental group did not use the ZTExApp as an e-diary.

“I didn't use the ZTEx smartphone app because it is quite troublesome as I need to press a few buttons to enter my ZTEx record in the app.” (Male, 67 years old)

“I seldom use the ZTExApp, as I can't see it even with presbyopia glasses.” (Male, 67 years)

“I have limited mobile data. I can only use Wi-Fi while I am at home. I can't access the ZTExApp when I go out.” (Female, 65 years)

“I am not quite familiar with using the internet.” (Male, 59 years old)

The participants liked the WhatsApp AIR ZTEx picture e-messages and commented that the e-messages were easy to understand.

“I like the WhatsApp ZTEx picture messages because they remind me to exercise.” (Male, 63 years old)

“The WhatsApp ZTEx picture messages can motivate me to do exercise”. (Male, 45 years old)

“When I go to the park to do morning exercises, I can use the ZTEx picture from WhatsApp as a guide to doing exercises.” (Male, 67 years old)

“The WhatsApp ZTEx picture messages remind me that I can do exercise while sitting. I don't have any excuse not to do exercise when I am working in my office.” (Male, 57 years old)

## Discussion

Our trial showed a moderate attrition rate (18.3%) and a good response rate on picture e-messages sent from an instant messaging platfrom (WhatsApp; 95%), but a low usage of ZTExApp. The experimental group reported a greater increase in walking time and a greater proportion of participants engaged in moderate PA, compared with the control group. Participants also reported high acceptability of an easy-to-do lifestyle integrating ZTEx and picture e-messages. Walking was the most common and acceptable exercise.

The good response rate might be related to using a readily available, affordable and prevalent platform (e.g., WhatsApp) to deliver the health-related picture e-messages. WhatsApp was the most common e-platform with an 84.3 % penetration rate in Hong Kong (44). Besides, we used picture e-messages that differed from the previous studies using text messages on PA only or PA combined with other information such as tobacco and diet messages (18, 20–28). A picture is worth a thousand words, and the ZTEx pictures should be easier for most participants to read than text messages, as some had presbyopia and older age group. Difficulties in reading texts in the ZTExApp, even with presbyopia glasses, might be a potential barrier to using the app for elderly participants. However, we had a low usage of ZTEx App which might relate to over 90% of our participants were aged 50 years or older. A review identified the top three most common barriers for elderly people using Apps that included (i) small font size, screen size, font type, buttons, color contrast, (ii) lack of experience and knowledge in technology, and (iii) menu with too many options, and navigation (45). Thus, it is important to consider the needs and requirements of elderly users in the design and development phase of a smartphone application to overcome the potential barriers to App use in future trials. Regarding the future intervention, proactive contact with participants to identify and overcome difficulties in using an app for the first 2 weeks might help familiarize with and enhance self-efficacy of using the App.

The high levels of acceptability and significant intervention effects favoring the experimental group might be related to the ease of integrating ZTEx into their daily life and the use of simple e-messages as exercise reminders to remind the participants to have regular exercise. Since walking was reported as the most common and acceptable PA, thus walking time significantly increased even though only three e-messages related to walking were sent to participants. Our findings were limited as the mechanism of the change was not investigated. For future studies, a larger scale RCT with longer follow-up, different e-messages with examples of ZTEx, and more time-points, plus qualitative data from in-depth interviews would help examine the mechanism or pathway for the PA behavior change and the sustainability of the longer-term effect. Participants should be asked how the messages they have received have led to the behavior changes.

The strengths of the study design were to use both qualitative and quantitative approaches to enrich the understanding of participants' feedback. We used a randomization trial design to minimize the selection bias and an active control group to enhance the rigor of the study. Health education on the risk factors of cardiovascular disease, the benefits of healthy eating and breathing exercises were chosen as active control information because the literature suggests that general health education sessions are reasonable activities and have been effective in retaining participants in the control group (46).

There were several limitations in our study. First, we measured perceptions and not actual knowledge in relation to ZTEx. Perceived knowledge may not reflect actual knowledge acquired and can be influenced by an individual's personality and self-perception (47). Perceived knowledge may be under- or over-estimated depending upon numerous factors in play at the time when completing the questionnaire. Second, we adopted a care bundle to use intervention with several components, but the effectiveness of each component was not examined. Examination of the effective components of our interventions would be a future direction for research and may allow refinement of the interventions for further trials and more specific interventions. Third, we used the last-observation-carried-forward imputation method for managing the missing data, which was a limitation because it might overestimate the precision compared to the multiple imputation method (48). Fourth, the self-reported IPAQ was used to measure PA, and recall bias might be greater in those with poor memory. The participants might have overestimated their PA when using the self-reported PA measures (49). Further studies should examine the PA using objective instruments to measure PA. Our data from Fitbit devices were limited, and future research should provide all participants with a reliable electronic device to measure PA (e.g., Fitbit or Actigraphy). Fifth, our participants were Chinese patients aged 50–69 years and had smartphones; the generalizability to other populations is uncertain, especially for non-Chinese people and people with low literacy and low socioeconomic status.

## Conclusion

In conclusion, this pilot study showed using a brief ZTEx face-to-face session with picture e-messages empowered patients with CHD to increase walking time and integrate PA into daily life. Future trials with a longer follow-up and a more user-friendly ZTExApp interface, are necessary to determine the effectiveness of the smartphone ZTEx intervention in other health-related outcomes.

## Data Availability Statement

The original contributions presented in the study are included in the article/Supplementary Material, further inquiries can be directed to the corresponding author/s.

## Ethics Statement

Ethics approval was approved by the Institutional Review Boards of the University of Hong Kong and the 3 HA clusters (IRB no. UW 16-363). The patients/participants provided their written informed consent to participate in this study.

## Author Contributions

NC contributed to the conception and design of the study, supervising data collection, data analysis, and manuscript drafting. AL contributed to the study's design, statistical analysis, and manuscript drafting. HC and KC contributed to the conception and design of the study and supervised data collection. AW contributed to the conception and design of the study and the recruitment strategy. DC contributed to the design study's analysis method and recruitment strategy. VC contributed to the conception and design of the study, editing and commenting on the manuscript drafts. YL and CY contributed to the data collection strategies and commenting and evaluating the manuscript. TL contributed to the conception and design of the study, drafting and editing the manuscript. All authors read and approved the final manuscript.

## Funding

This study was supported by the Health and Medical Research Fund (HMRF) of the Food and Health Bureau of the Hong Kong SAR Government (Grant no. 1415 1111).

## Conflict of Interest

The authors declare that the research was conducted in the absence of any commercial or financial relationships that could be construed as a potential conflict of interest.

## Publisher's Note

All claims expressed in this article are solely those of the authors and do not necessarily represent those of their affiliated organizations, or those of the publisher, the editors and the reviewers. Any product that may be evaluated in this article, or claim that may be made by its manufacturer, is not guaranteed or endorsed by the publisher.
