# Supplementary material for: Feasibility and Potential Effectiveness of a Smartphone Zero-Time Exercise Intervention for Promoting Physical Activity and Fitness in Patients With Coronary Heart Disease: A Pilot Randomized Controlled Trial
Source: Front Public Health. 2022 Jul 14;10:865712. doi: 10.3389/fpubh.2022.865712 (PMC9330491; doi:10.3389/fpubh.2022.865712)
Supplement: Supplementary file 1 [file Table_1.pdf]

Supplementary Table I The brief individual face-to-face session for the experimental and control groups

**The experimental group (n=70)**	
**Participants received brief ZTEx-related information (15 min)**	
**Steps:**	**Aims:**
1. Briefly discuss with the participants about the risk factors related to coronary heart disease (CHD), and ways of reducing the risks (e.g. increase physical activity)<br>2. Introduce the concepts of ZTEx and the health benefits of performing ZTEx.<br>3. Demonstrate the examples of different poses of ZTEx and invite the participants to perform the ZTEx together. e.g. hand grip exercise, shoulder raise, pedalling both legs, raising both heels.<br>4. Instruct the participants to use the ZTExApps for further information on and examples of ZTEx.<br>5. Suggest participants to perform at least 30 mins per day and record the PA time in the e-dairy in the ZTEx App.	• To increase participants' risk perception on CHD<br>• To enhance positive and realistic outcome expectancies.<br>• To increase the participants' action self-efficacy of being active<br>• To further enhance knowledge on ZTEx.<br>• To facilitate goal setting and self-monitoring on doing PA, including ZTEx.
**The control group (n=69)**	
**Participants received brief healthy eating and breathing exercise information (15 min)**	
1. Briefly discuss with the participants about the risk factors related to coronary heart disease (CHD), and ways of reducing the risks (e.g. healthy eating with low sugar and salt consumption).<br>2. Introduce healthy eating (e.g low fat, salt & sugar consumption) and its health benefits to promote cardiovascular health.<br>3. Suggest ways of eating healthy (e.g. taking at least 2 servings of fruit and 3 servings of vegetables every day).<br>4. Introduce the benefit of practicing breathing exercises (e.g. relaxation) and invite the participants to perform breathing exercises together.<br>5. Instruct the control participants to perform breathing exercises for at least 30 mins every day.	• To increase the participants' risk perception on CHD<br>• To enhance positive and realistic outcome expectancies from healthy eating<br>• To increase the participants' action self-efficacy of eating healthy
