# Supplementary material for: Feasibility and Potential Effectiveness of a Smartphone Zero-Time Exercise Intervention for Promoting Physical Activity and Fitness in Patients With Coronary Heart Disease: A Pilot Randomized Controlled Trial
Source: Front Public Health. 2022 Jul 14;10:865712. doi: 10.3389/fpubh.2022.865712 (PMC9330491; doi:10.3389/fpubh.2022.865712)
Supplement: Supplementary file 2 [file Table_2.pdf]

Supplementary Table 2 The 28 anti-inertia reminder (AIR) picture e-messages via WhatsApp for the experimental and control groups

e-Message No.	Experimental group (received ZTEx/physical activity picture intervention)	Experimental group AIR picture e-messages	Control group (received healthy eating and breathing picture interventions)	Control group AIR picture e-messages	Goals
	**Week 1**				
e-Message 1	**Picture e-message description:**<br>***Set your Goals & record your progress***<br>• First, set your goals for performing ZTEx, and record your progress.<br>• Persevere, and you will realise the benefits of doing exercise.<br>• Keep doing ZTEx; you will look good and feel good.<br>• Remember to do it with your family members.	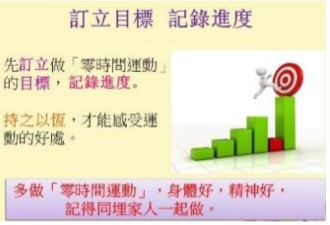	**Picture e-message description:**<br>***Perform breathing exercises (BE) &***<br><br>***Record your time spent on physical activities in your dairy***	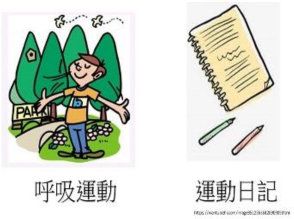	• To motivate the participants to perform taught behaviors.
e-Message 2	**Picture e-message description:**<br>• Please share with your family: Handgrip strength and health<br>• Increasing 5 kg handgrip strength<br>– can reduce the risk of cardiovascular disease by 7%<br>– can reduce stroke mortality by 9%.	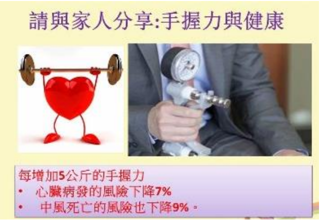	**Picture e-message description:**<br>***Deep breathing exercise***<br>*Advantages:*<br>• Reduce stress and can help with insomnia.	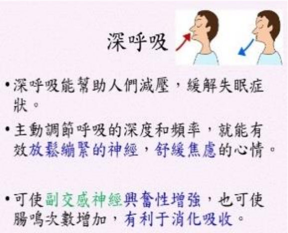	• To increase the participants' risk perception of CHD, and<br>• To motivate them to increase PA.
e-Message 3	**Picture e-message description:**<br>***Squeeze the soft plastic star to improve handgrip strength***	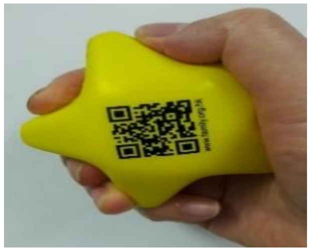	**Picture e-message description:**<br>***Healthy eating***<br>• Pyramid Healthy Eating Food	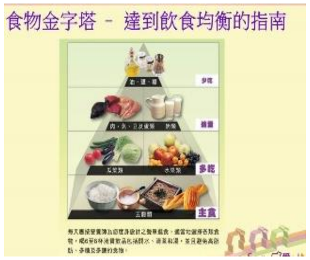	• To increase the participants' action self-efficacy

e-Message 4	**Picture e-message description:**<br>***Handgrip strength – holding a teaspoon***<br>• Using a hand grip strengthener to hold a teaspoon at one end<br>• Start timing. Try to keep the position.<br>• When the spoon falls, record the time of holding the spoon. Use seconds as the unit.	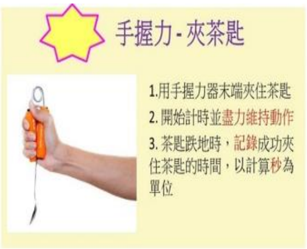	**Picture e-message description:**<br>***Healthy eating***<br>• Healthy meat serving size per	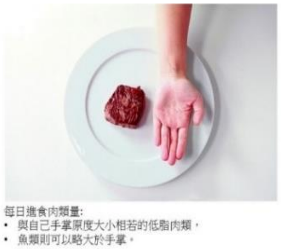	• To increase the participants' action self-efficacy
e-Message 5	**Picture e-message description:**<br>***Raise the buttock and the upper part of the body***<br>• Lie down flat with face-up, bend your knees, and slowly raise the upper part of your body and your buttock.<br>• Use your shoulders as the supporter, with soles touching the ground.<br>• Keep the shoulders, buttock and knees in a straight line	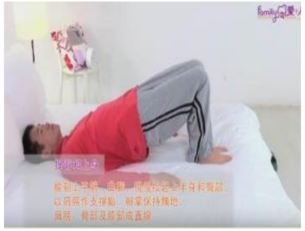	**Picture e-message description:**<br>***Healthy diet***<br>• Taking oats with a description of the health benefits	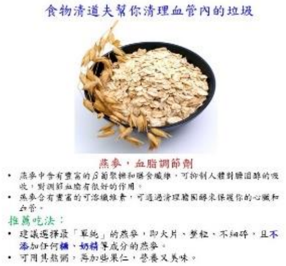	• To motivate the participants to perform taught behaviors.
e-Message 6	**Picture e-message description:**<br>***No more sitting all day long***<br>***Let's brisk walk and be strong***	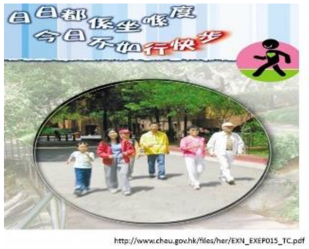	**Picture e-message description:**<br>***Healthy eating***<br>• Serving food portion sizes promotes plant-based proteins.	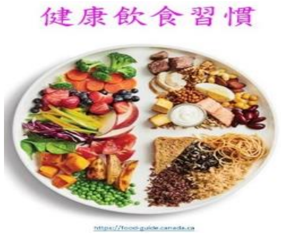	• To increase the participants' action self-efficacy
e-Message 7	**Picture e-message description:**<br>***Holding your shopping bags away from your body.***	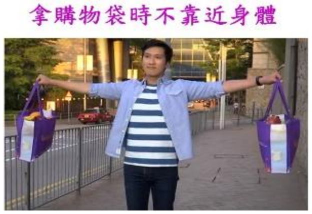	**Picture e-message description:**<br>***Healthy diet***<br>• Benefits of taking celery stalks	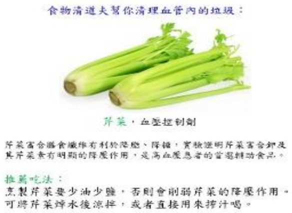	• To increase the participants' action self-efficacy

Week 2 to week 6					
e-Message 8	**Picture e-message description:**<br>**(same as e-message 1)**	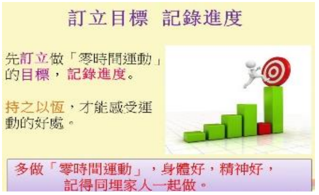	**Picture e-message description:**<br>***Breathing exercise***<br>• Promote relaxation	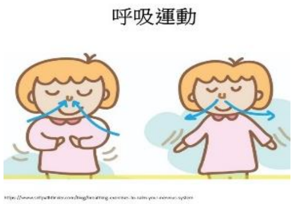	• To motivate the participants to perform taught behaviors.
e-Message 9	**Picture e-message description:**<br>(same as e-message 4)	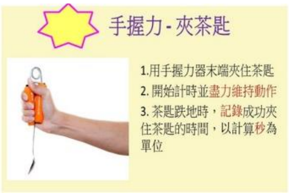	**Picture e-message description:**<br>***Healthy eating***<br>• Healthy Eating Food Pyramid	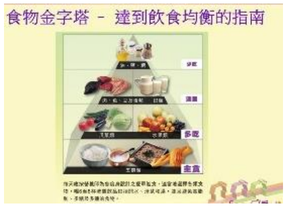	• To increase the participants' action self-efficacy
e-Message 10	**Picture e-message description:**<br>***Doing exercise while sitting -- 1***	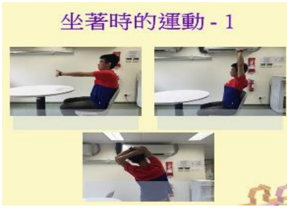	**Picture e-message description:**<br>***Healthy eating***<br>• Chinese soup picture with the recipe.	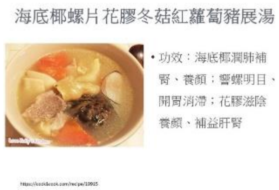	• To increase the participants' action self-efficacy
e-Message 11	**Picture e-message description:**<br>***Doing exercise while sitting -- 2***	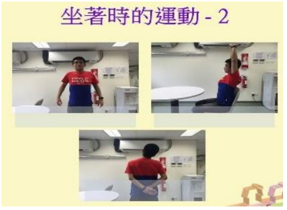	**Picture e-message description:**<br>***Healthy eating***<br>• Low calorie and fat beef soup noodle (restaurant food).	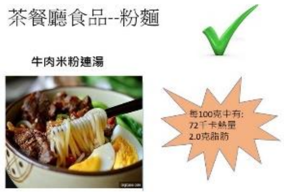	• To increase the participants' action self-efficacy
e-Message 12	**Picture e-message description:**<br>***Doing exercise while sitting -- 3***	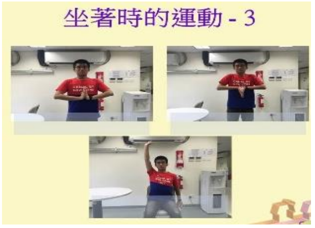	**Picture e-message description:**<br>***Healthy eating***<br>• Chinese soup with the recipe.	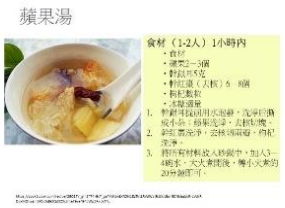	• To increase the participants' action self-efficacy

e-Message 13	**Picture e-message description:**<br>(same as e-message 5)	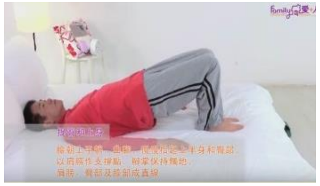	**Picture e-message description:**<br>***Breathing exercise***<br>• Promote relaxation	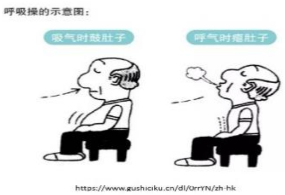	• To motivate the participants to perform taught behaviors.
e-Message 14	**Picture e-message description:**<br>(same as e-message 6)	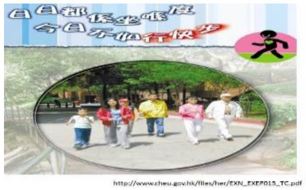	**Picture e-message description:**<br>***Healthy eating***<br>• Low calorie and fat fish soup noodles (restaurant food).	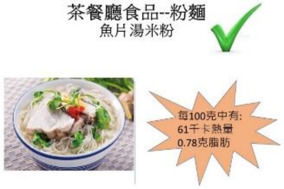	• To increase the participants' action self-efficacy
e-Message 15	**Picture e-message description:**<br>(same as e-message 7)	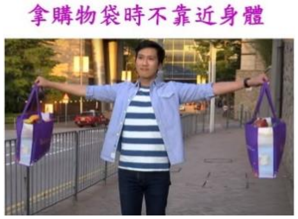	**Picture e-message description:**<br>***Healthy eating***<br>• Daily sugar, salt, and fat intake limit.	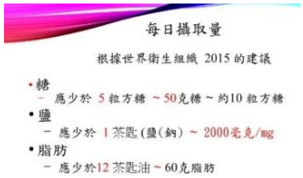	• To increase the participants' action self-efficacy
e-Message 16	**Picture e-message description:**<br>***Climbing stairs fast***	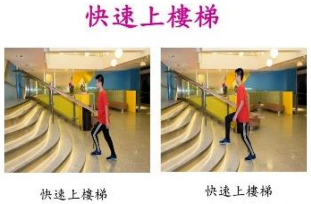	**Picture e-message description:**<br>***Unhealthy eating***<br>• High calorie, salt and fat pork ribs and chicken feet steam rice (restaurant food).	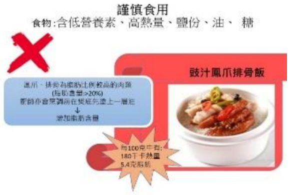	• To increase the participants' action self-efficacy
e-Message 17	**Picture e-message description:**<br>***Push up from the chair***<br>*Procedure:*<br>• Sit on a chair. Move the buttock and feet forward. Use your arm's strength to support your body.<br>• Use the upper arms to raise and lower the body.<br>*Advantages:*<br>• Train muscles of the upper extremity (behind arms, shoulders, chest)	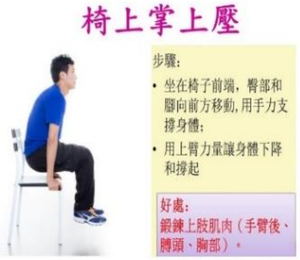	**Picture e-message description:**<br>***Unhealthy eating***<br>• High calorie, salt and fat curry beef rice (restaurant food).	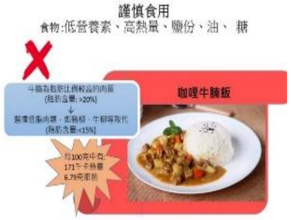	• To increase the participants' action self-efficacy

e-Message 18	**Picture e-message description:**<br>***Cross Knee Stretch***<br>*Procedure:*<br>• Cross one knee over the other foot<br>• Slightly lean forward, with neck and waist kept in a straight line.<br>• Keep leaning until the buttock feels a little bit tense.<br>*Advantages:*<br>• Substantial bend of the hip joint of the buttock,,<br>• soothe the tight muscle of the buttock,<br>• help in relieving low back pain.	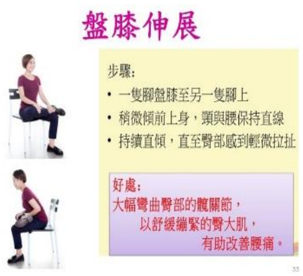	**Picture e-message description:**<br>***Breathing exercise***<br>• Promote relaxation	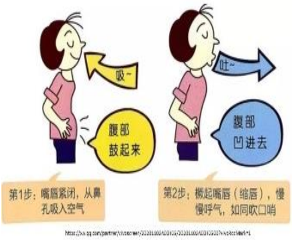	• To increase the participants’ action self-efficacy
e-Message 19	**Picture e-message description:**<br>***Strengthening the knee muscles***<br>*Procedure:*<br>• Hold the chair with both arms, raise the legs<br>• Keep the legs up in the air, keep the posture.<br>*Advantages:*<br>• Legs raised when sitting can train muscles of the abdomen (rectus abdominis) and the thigh (quadriceps).<br>• Strengthen the knee muscles; it can reduce falls	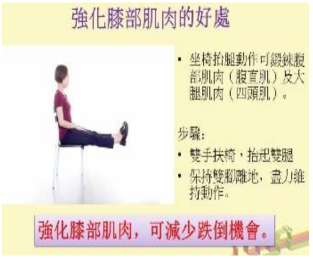	**Picture e-message description:**<br>***Healthy eating***<br>• Low-calorie ham macaroni soup (restaurant food).	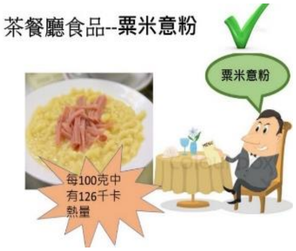	• To increase the participants’ action self-efficacy
e-Message 20	**Picture e-message description:**<br>***Small Knee Bend***<br>Procedure:<br>• Slightly bend the knees while standing, with the upper part of the body remaining upright.<br>• Note: the knees should not go beyond the tips of the toes.	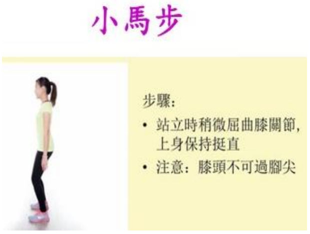	**Picture e-message description:**<br>***Healthy eating***<br>• Compare the sugar level of kiwifruit and grapes.	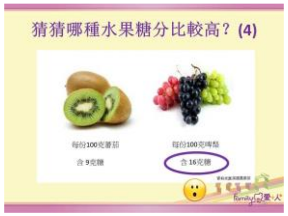	• To increase the participants’ action self-efficacy

e-Message 21	**Picture e-message description:**<br>***Standing on Tiptoe***<br>• The balancing power needs the coordination of various body systems, including muscles, the visual, nervous system and inner ear.<br>• The balancing power will decrease as age increases.<br>*Advantages:*<br>Standing on tiptoe can train the muscles of the ankle and the calf. It can also prevent varicose veins.	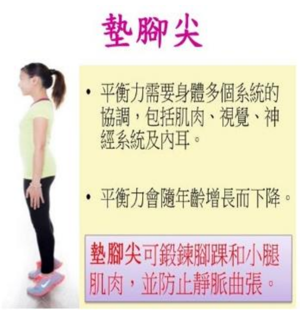 	**Picture e-message description:**<br>***Breathing exercise***<br>• Promote relaxation	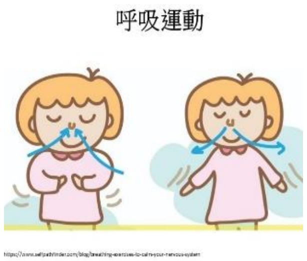 	• To increase the participants' action self-efficacy
e-Message 22	**Picture e-message description:**<br>(same as e-message 16)	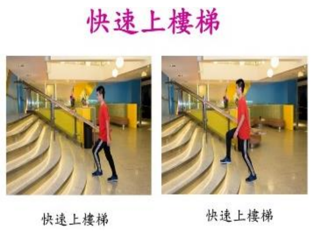 	**Picture e-message description:**<br>***Healthy eating***<br>• Compare the sugar level of red bean ice drink, lemon ice tea and ice bubble milk tea.	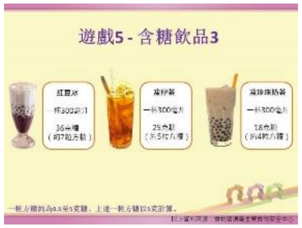 	• To increase the participants' action self-efficacy
**Week 7 to week 12**					
e-Message 23	**Picture e-message description:**<br>**(same as e-message 1)**	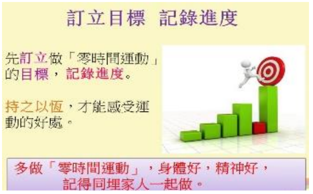 	**Picture e-message description:**<br>***Healthy eating***<br>• Chinese soup with the recipe.	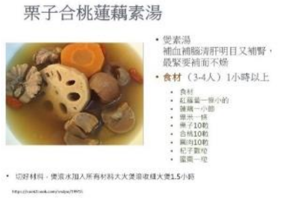 	• To motivate the participants to perform taught behaviors.
e-Message 24	**Picture e-message description:**<br>(same as e-message 4)	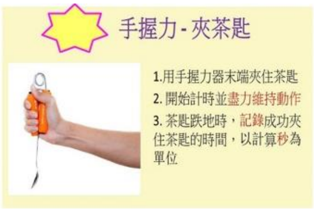 	**Picture e-message description:**<br>***Healthy eating***<br>• Compare the sugar level of hot coffee and milk tea.	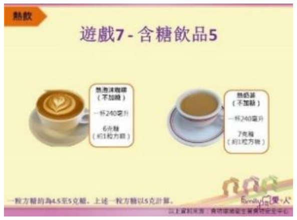 	• To increase the participants' action self-efficacy

e-Message 25	**Picture e-message description:**<br>***Weightlifting Test***<br>• Mimic the action of rope skipping, skip in place. Keep a good breathing rhythm.<br>• Record the number of weightlifting tests in 20 seconds; use seconds as the unit.	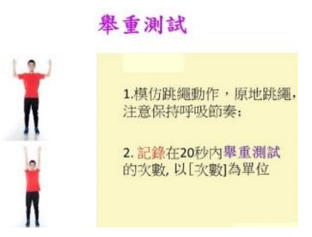	**Picture e-message description:**<br>***Breathing exercise***<br>• Promote relaxation	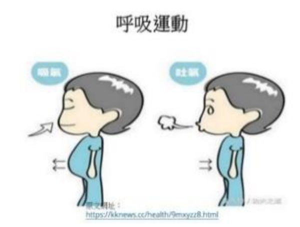	• To increase the participants' action self-efficacy
e-Message 26	**Picture e-message description:**<br>***Straight Arm Raise***<br>*Procedure*:<br>• Raise both arms until they form a straight line with the shoulders (beginning position).<br>• Raise both arms until they are perpendicular to the shoulders, and stretch your arms (ending position).<br>*Advantages:*<br>• Train upper arm and back muscles.	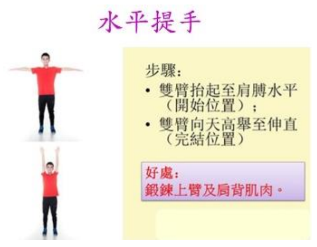	**Picture e-message description:**<br>***Unhealthy eating***<br>• High calorie, salt and fat fried noodle (restaurant food).	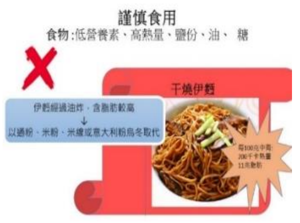	• To increase the participants' action self-efficacy
e-Message 27	**Picture e-message description:**<br>***Missing Chair against the Wall***<br>*Procedure:*<br>• With your back against the wall, hands at the two sides, keep the posture.<br>*Advantages*:<br>• As long as there is no inflammation or pain in the joint, doing exercise is the best prescription for protecting the knee joint and strengthening bone density.	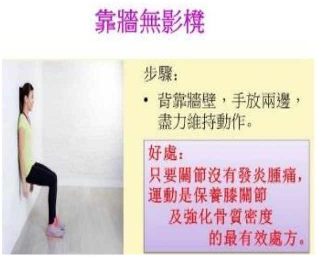	**Picture e-message description:**<br>***Unhealthy eating***<br>• High calorie, salt and fat roast pork rice (restaurant food).	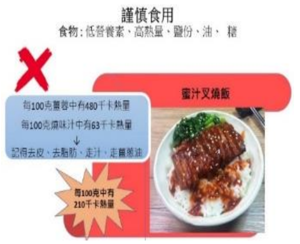	• To increase the participants' action self-efficacy.
e-Message 28	**Picture e-message description:**<br>(same as e-message 7)	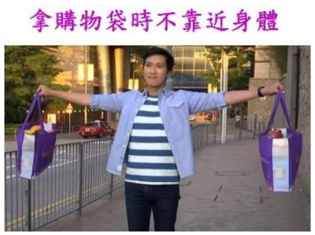	**Picture e-message description:**<br>***Healthy eating***<br>• Serving food portion sizes promotes plant-based proteins.	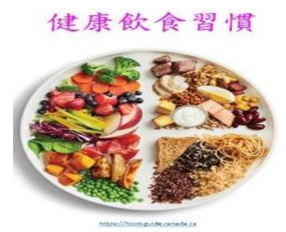	• To increase the participants' action self-efficacy

Remarks A brief description of the contents for the control group is included herein. Full English translation will be provided upon request.
