# Supplementary material for: Feasibility and Potential Effectiveness of a Smartphone Zero-Time Exercise Intervention for Promoting Physical Activity and Fitness in Patients With Coronary Heart Disease: A Pilot Randomized Controlled Trial
Source: Front Public Health. 2022 Jul 14;10:865712. doi: 10.3389/fpubh.2022.865712 (PMC9330491; doi:10.3389/fpubh.2022.865712)
Supplement: Supplementary file 3 [file Table_3.pdf]

Supplementary Table 3: The semi-structured individual interview guide

1. What are your expected benefits from doing ZTEx and having regular physical activity?<br>2. What are the barriers and facilitators to doing ZTEx and having regular physical activity?<br>3. What are your comments on the picture e-messages and ZTExApp?<br>4. What are the barriers and facilitators of reading the picture e-messages and using the ZTExApp?<br>5. Did the picture e-messages and/or ZTExApp help improve your physical activity habit?<br>6. What are your perceptions on this smartphone ZTEx intervention such as its useability and acceptability?<br>7. What are your suggestions on the smartphone ZTEx intervention for further improvement?
