# Supplementary material for: Feasibility and Potential Effectiveness of a Smartphone Zero-Time Exercise Intervention for Promoting Physical Activity and Fitness in Patients With Coronary Heart Disease: A Pilot Randomized Controlled Trial
Source: Front Public Health. 2022 Jul 14;10:865712. doi: 10.3389/fpubh.2022.865712 (PMC9330491; doi:10.3389/fpubh.2022.865712)
Supplement: Supplementary file 4 [file Table_4.pdf]

Supplementary Table 4   Characteristics of the participants who completed the 12-week follow-up (n=113)

	Experimental group (n=56)	Control group (n=57)	P-value
	**n (%)**	**n (%)**	
**Sex**			
Female	19 (33.9)	13 (22.8)	0.19
Male	37 (66.1)	44 (77.2)	
**Age, years**[#]	60.1 ± 6.6	60.4 ± 6.6	0.83
**Body Mass Index (kg/m^2)** [#]	25.9 ± 3.4	27.1 ± 3.3	0.06
**Marital status**			
Single/divorced	9 (16.1)	13 (22.8)	0.37
Married	47 (83.9)	44 (77.2)	
**Working status**			
Retired, unemployed, or working part-time	30 (53.6)	33 (57.9)	0.64
Working full-time	26 (46.4)	24 (42.1)	
**Smoking status**			
Never smokers	38 (67.9)	35 (61.4)	0.64
Smokers and ex-smokers	17 (30.4)	19 (33.3)	
Missing data	1 (1.8)	3 (5.3)	

[#] Presented as mean ± SD
