# Supplementary material for: Feasibility and Potential Effectiveness of a Smartphone Zero-Time Exercise Intervention for Promoting Physical Activity and Fitness in Patients With Coronary Heart Disease: A Pilot Randomized Controlled Trial
Source: Front Public Health. 2022 Jul 14;10:865712. doi: 10.3389/fpubh.2022.865712 (PMC9330491; doi:10.3389/fpubh.2022.865712)
Supplement: Supplementary file 5 [file Table_5.pdf]

Supplementary Table 5 Exercise self-efficacy and intention, physical fitness, perceived happiness and health, and quality of life of the experimental and control groups at baseline and 12-week follow-up by per-protocol analysis (n=113)

	Experimental group (n=56)				Control group (n=57)				Between-group comparison at 12-week FU		
	Baseline	12-week follow-up	Within-group difference		Baseline	12-week follow-up	Within-group difference				
	Mean ± SD	Mean ± SD	P-value	Cohen's d	Mean ± SD	Mean ± SD	P-value	Cohen's d	Adjusted mean difference (95% CI)	P-value	Cohen's d
**Exercise self-efficacy and intention**											
Knowledge of ZTEX	7.1 ± 2.7	7.8 ± 1.7	0.07	0.25	---	---	---	---	---	---	---
Self-efficacy of doing ZTEx	6.7 ± 2.2	6.7 ± 1.8	0.91	0.02							
Expectancy on ZTEx	7.2 ± 2.2	7.6 ± 1.9	0.24	0.16	---	---	---	---	---	---	---
Plan of doing ZTEx	7.1 ± 2.3	6.9 ± 2.0	0.51	-0.09	---	---	---	---	---	---	---
**Physical fitness**											
**Body composition**											
Bodyweight, Kg	70.1 ± 12.0	70.1 ± 11.9	0.98	0.00	74.3 ± 11.7	74.1 ± 11.6	0.49	-0.02	0.08 (-0.57, 0.74)	0.81	0.04
Body fat, %	30.3 ± 5.8	29.9 ± 6.4	0.30	-0.14	29.2 ± 5.6	29.0 ± 5.5	0.75	-0.04	-0.24 (-1.30, 0.81)	0.65	-0.08
Waist circumference, cm	92.4 ± 10.2	91.8 ± 9.8	0.13	-0.06	96.3 ± 10.2	96.4 ± 8.6	0.84	0.02	-1.40 (-2.96, 0.17)	0.08	-0.32
**Muscle strength**											
Handgrip test (right), kg	30.2 ± 20.7	27.7 ± 7.8	0.32	-0.16	28.4 ± 9.5	29.5 ± 10.2	0.13	0.11	-1.36 (-3.97, 1.26)	0.31	-0.18
Handgrip test (left), kg	26.9 ± 9.0	26.9 ± 7.9	0.98	0.00	26.8 ± 9.7	27.2 ± 9.9	0.60	0.04	-0.02 (-1.89, 1.85)	0.98	0.00
30-sec chair stand test, no. of stands	15.6 ± 7.1	17.7 ± 6.5	0.01*	0.30	15.2 ± 4.3	15.9 ± 5.2	0.20	0.16	1.32 (-0.46, 3.10)	0.15	0.27
**Perceived happiness and health**											
Perceived happiness	6.8 ± 1.7	7.3 ± 1.7	<0.001***	0.33	7.2 ± 2.0	7.4 ± 1.9	0.39	0.09	0.24 (-0.22, 0.70)	0.30	0.19
Perceived health	6.3 ± 2.0	6.2 ± 1.7	0.80	-0.03	6.2 ± 1.8	6.4 ± 2.1	0.44	0.13	-0.22 (-0.87, 0.43)	0.51	-0.12
**Quality of life**											
Physical health	14.4 ± 1.5	15.4 ± 1.9	<0.001***	0.60	14.2 ± 2.1	15.0 ± 2.1	0.005**	0.37	0.36 (-0.30, 1.03)	0.28	0.20
Psychological	13.0 ± 1.5	15.3 ± 2.3	<0.001***	1.18	12.6 ± 2.0	14.8 ± 2.5	<0.001***	0.96	0.27 (-0.55, 1.09)	0.52	0.12
Social Relationships	14.9 ± 2.1	15.0 ± 2.2	0.63	0.07	14.9 ± 2.7	14.1 ± 2.2	0.01*	-0.32	0.93 (0.22, 1.65)	0.01[#]	0.47
Environment	15.3 ± 1.8	15.6 ± 1.8	0.26	0.16	14.9 ± 2.3	14.8 ± 2.1	0.62	-0.06	0.61 (-0.02, 1.24)	0.06	0.35

ZTEx: Zero-time exercise refers to simple strength- and stamina-enhancing physical activity

Exercise self-efficacy and intention regarding ZTEx using a scale of 0-10 for each question; higher scores indicate better outcomes.

Perceived happiness and health using a scale of 0-10; higher scores indicate better outcomes.

Quality of life using a WHO Quality of Life-Short Form (WHOQOL-BREF) with a 5-point Likert scale, higher scores indicate better quality of life.

Within-group comparison during the 12-week follow-up was increased by paired sample t-test; *P <0.05, *** P <0.001

The between-group comparison was done by linear mixed model, adjusted for age, sex and baseline values; [#]P <0.05

Effect size (Cohen’s d): small, 0.2; moderate, 0.5; large, 0.8
