# Supplementary figures and images for: Feasibility and Potential Effectiveness of a Smartphone Zero-Time Exercise Intervention for Promoting Physical Activity and Fitness in Patients With Coronary Heart Disease: A Pilot Randomized Controlled Trial
Source: Front Public Health. 2022 Jul 14;10:865712. doi: 10.3389/fpubh.2022.865712 (PMC9330491; doi:10.3389/fpubh.2022.865712)

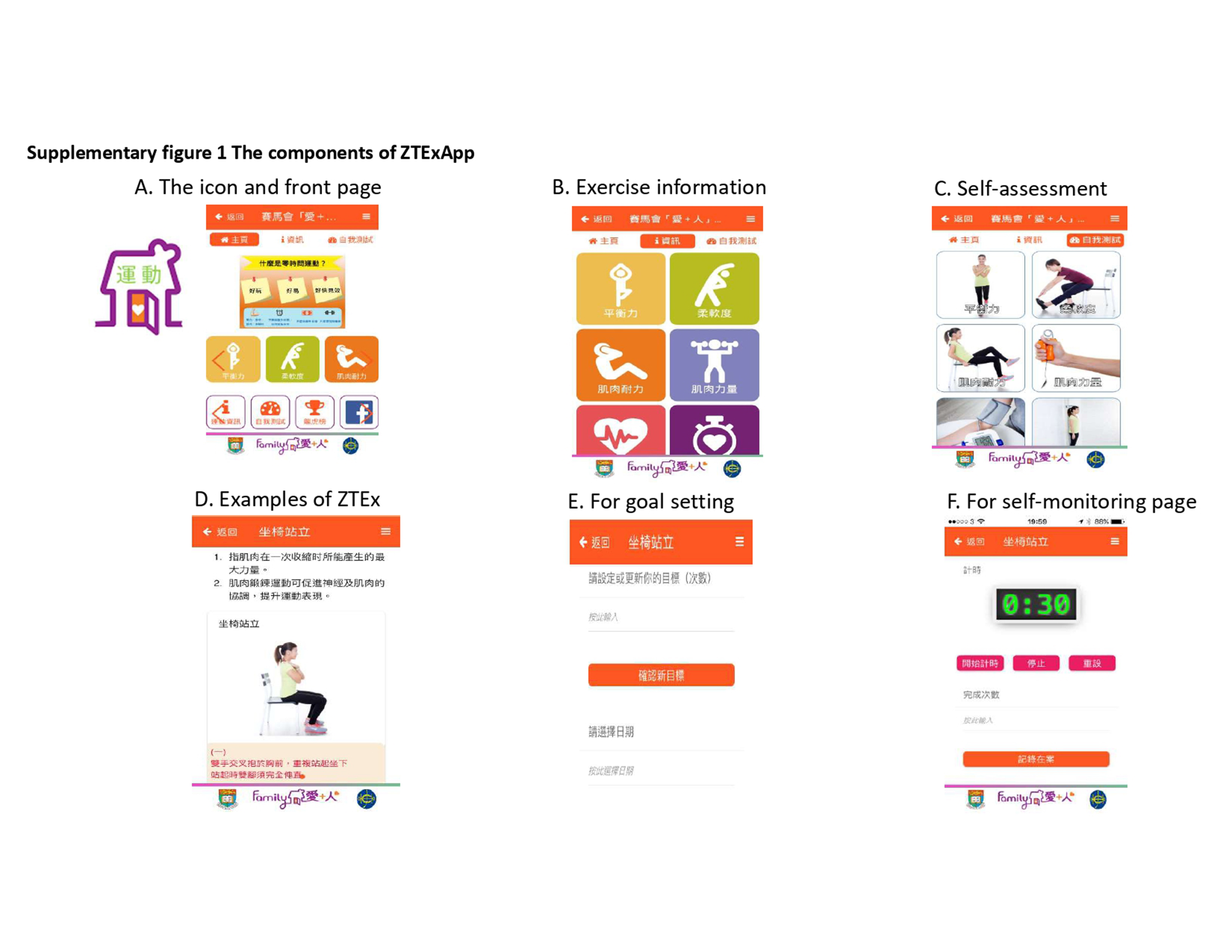

Supplement: Supplementary file 6 [file Image_1.jpeg]

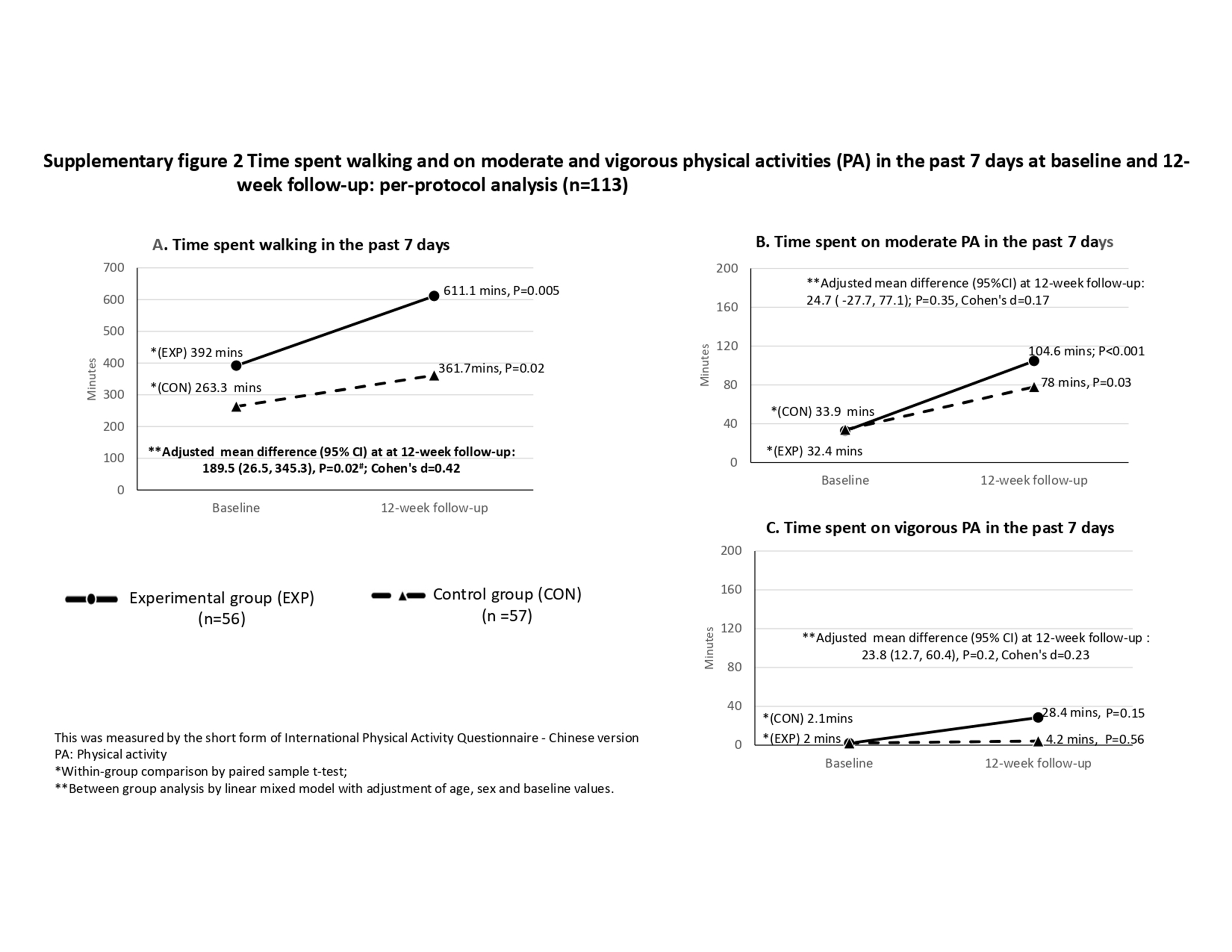

Supplement: Supplementary file 7 [file Image_2.jpeg]

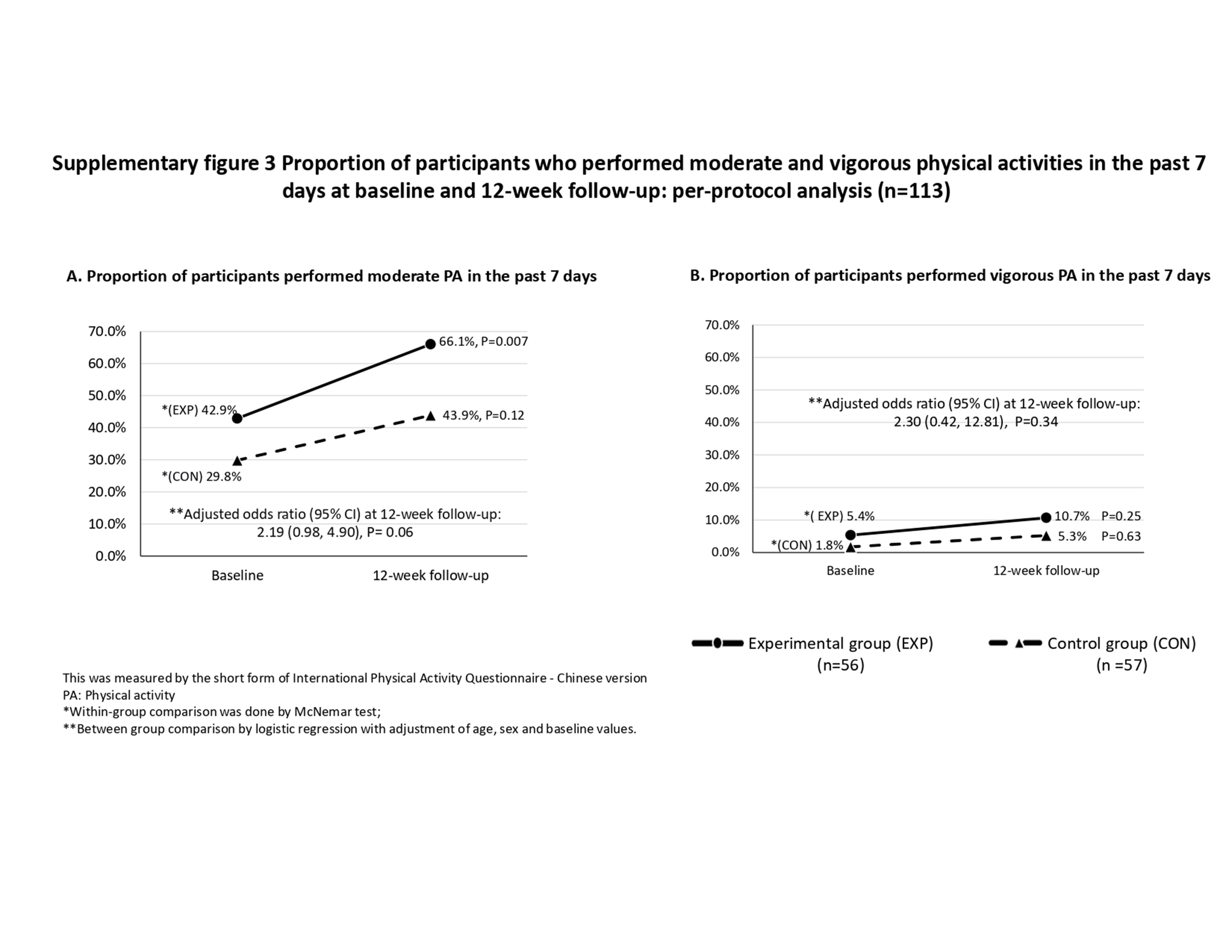

Supplement: Supplementary file 8 [file Image_3.jpeg]
